# The complete mitochondrial genome and phylogenetic position of *Orthaga aenescens* Moore, 1888 (Lepidoptera: Pyralidae)

**DOI:** 10.1080/23802359.2026.2722723

**Published:** 2026-08-24

**Authors:** Laiyou Wang, Jiali Liu, Shuyu Hui, Hua Rong, Yu Wang

**Affiliations:** ^a^ School of Biological and Chemical Engineering, Nanyang Institute of Technology, Nanyang, China; ^b^ College of Bioscience and Engineering, Jiangxi Agricultural University, Nanchang, China

**Keywords:** *Orthaga aenescens*, Pyralidae, mitochondrial genome, phylogenetic position

## Abstract

The genus *Orthaga* includes several agricultural and forestry pests. However, only a few complete mitogenomes have been reported for *Orthaga*. To address this gap, we sequenced and characterized the complete mitogenome of *Orthaga aenescens* Moore, 1888. The circular mitogenome of *O. aenescens* is 15,160 bp in length and comprises 13 protein-coding genes (PCGs), 22 transfer RNA genes (tRNAs), 2 ribosomal RNA genes (rRNAs), and a control region. The base composition shows a strong bias toward adenine and thymine, with 80.7% of the bases being A or T, while the AT-skew is slightly negative at −0.031. Phylogenetic analysis suggests that among the sequenced Pyralidae mitochondrial genomes, *O. aenescens* is most closely related to *O. euadrusalis* and *Locastra muscosalis*, supporting its placement within the subfamily Epipaschiinae.

## 1. Introduction

The genus *Orthaga* (Lepidoptera: Pyralidae) is widely distributed across the Oriental, Palearctic, and Australian regions, and includes numerous economically significant pests of agricultural and forestry crops. Among them, *O. olivacea*, *O. achatina*, and *O. euadrusalis* are notorious for causing severe defoliation and yield losses in plantations of Lauraceae (e.g. *Cinnamomum camphora*, *C. cassia*, *Lindera glauca*), Fagaceae (e.g. *Castanea mollissima*, *Lithocarpus glaber*), and Anacardiaceae (e.g. *Rhus chinensis*, *Mangifera indica*) (Yang et al. 2020; Liu et al. 2021a, 2021b). Despite their economic importance, accurate species identification within *Orthaga* remains challenging due to morphological similarity among adults and overlapping larval host plant ranges. For example, the common name ‘camphor webworm’ has been applied inconsistently in the literature, sometimes referring to *O. olivacea* and other times to *O. achatina* (Yang et al. 2020; Liu et al. 2021a), leading to confusion in pest management and biological control programs.

Traditional taxonomy based on external morphology and genitalia often fails to reliably distinguish closely related *Orthaga* species, especially when only immature stages are available (Mutanen et al. 2010). Molecular markers, particularly mitochondrial DNA sequences, have proven effective for species delimitation and phylogenetic reconstruction in Lepidoptera. Its maternal inheritance, lack of recombination, relatively rapid evolutionary rate, and conserved gene content make it an ideal tool for resolving taxonomic ambiguities and inferring evolutionary relationships at various taxonomic levels (Dong et al. 2021). To date, complete mitogenomes have been reported for several pyralid species (Yang et al. 2023; Wang et al. 2024), but representatives of the genus *Orthaga* remain scarce, limiting our understanding of their phylogenetic positions and genetic diversity.

In this study, we sequenced and characterized the complete mitochondrial genome of *Orthaga aenescens* Moore, 1888, a species with limited genomic information despite its ecological significance. Additionally, a maximum-likelihood (ML) phylogenetic analysis based on the 13 protein‑coding genes was performed to clarify the phylogenetic position of *O. aenescens* within Pyralidae. This work expands the mitochondrial genomic resources for the genus *Orthaga* and provides essential molecular data for species identification and future phylogenetic studies of pyralid pests.

## 2. Materials and methods

An adult of *O. aenescens* was collected using a light trap in August 2022 from Datangwan (26°21′N, 108°09′E), Leishan County, Guizhou Province, China. The specimen was deposited in the Entomological Museum of the College of Bioscience and Engineering, Jiangxi Agricultural University, under the voucher number MT007 (contact person: Hua Rong, email: ronghua@jxau.edu.cn).

**Figure 1.**
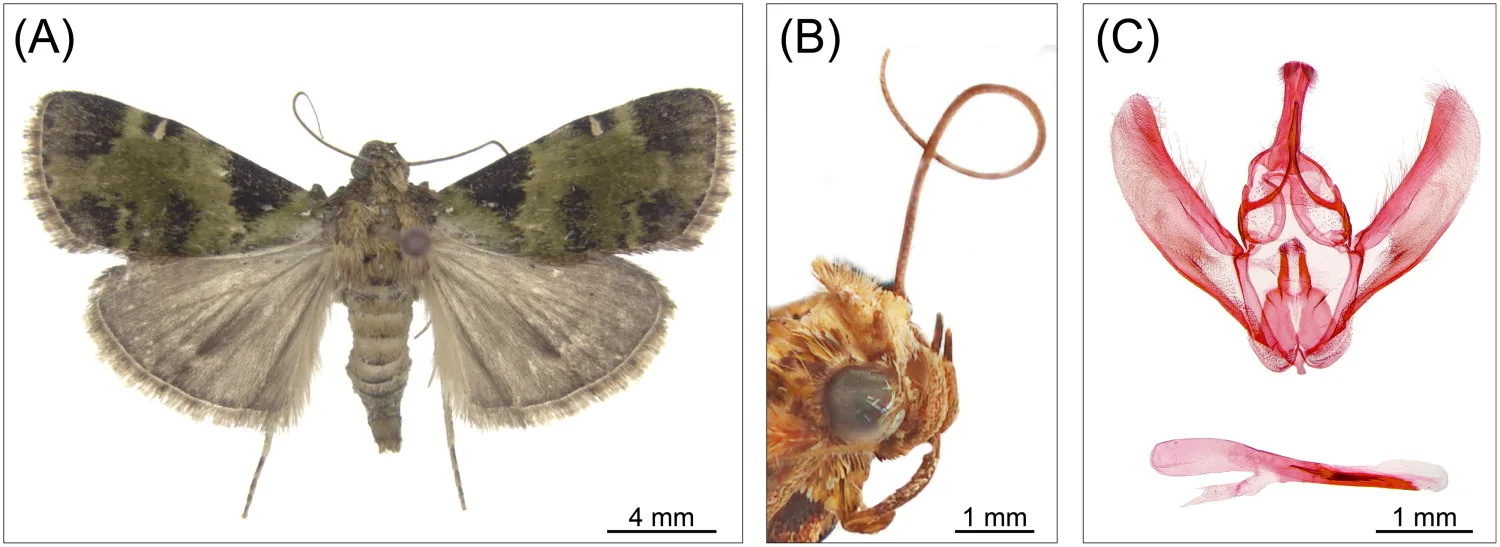
Morphological features of *O. aenescens*. (A) Adult male in dorsal view. (B) Male antenna showing a short scape extension. (C) Aedeagus showing a bundle of spiny cornuti. Photographs by Hua Rong.

The species *O. aenescens* can be clearly distinguished from other species of the genus *Orthaga* by the following combination of characters. The forewing is predominantly green, with a black inverted triangular spot at the base of the costa (Figure 1A). The male antenna bears a short scape extension (Figure 1B), and the aedeagus bears a bundle of spiny cornuti (Figure 1C).

The entire specimen was preserved in absolute ethanol and stored at −20 °C for subsequent DNA extraction. Total DNA was extracted from thoracic muscle tissue of a single adult using the HiPure Insect DNA Kit (Magen, China) following the manufacturer’s protocol. Approximately 0.2 μg of total DNA was subjected to random fragmentation by sonication to an average size of 350 bp, after which the DNA fragments were end-polished, A-tailed, and ligated to Illumina adapters. The ligated products were subsequently PCR-amplified and purified using the AMPure XP system (Beverly, USA). Library quality was assessed on an Agilent 5400 system (Agilent, USA) and quantified using qPCR, after which qualified libraries were pooled and sequenced on an Illumina NovaSeq 6000 platform with a PE150 strategy at Novogene Bioinformatics Technology Co., Ltd. (Beijing, China). Finally, a total of 6.71 Gb raw data were generated, and clean paired-end reads were obtained using fastp v0.23.2 (Chen 2023). Subsequently, the mitogenome was assembled using SPAdes v3.14 (Bankevich et al. 2012), annotated with MITOS2 (Donath et al. 2019), and then manually adjusted. The circular genome map was drawn with the program Proksee v6.0.2 (Grant et al. 2023). Nucleotide composition was calculated using PhyloSuite v1.2.2 (Zhang et al. 2020). Strand asymmetry was computed using the following equations: AT-skew = [A−T]/[A + T] and GC-skew = [G−C]/[G + C] (Perna & Kocher 1995).

To investigate the phylogenetic position of *O. aenescens*, 23 mitogenomes of the closest published species were downloaded based on the blasting results in GenBank. *Spodoptera litura* and *Spodoptera frugiperda* were selected as the outgroups. The nucleotide sequences of the 13 PCGs were each translated into amino acid sequences, individually aligned using MAFFT as implemented in PhyloSuite v1.2.2 (Zhang et al. 2020), and then converted back to nucleotide alignments to prevent the influence of AT bias in the mitogenome nucleotide sequence data. Gaps and ambiguous sites were removed using Gblocks v0.91b (Castresana 2000), and individual alignments were concatenated using PhyloSuite v1.2.2 (Zhang et al. 2020). The ML tree was reconstructed using IQ-TREE v2.4.0 (Minh et al. 2020) as implemented in PhyloSuite v1.2.2. The concatenated alignment was analyzed under a single-partition framework. The optimal nucleotide substitution model was automatically selected using ModelFinder based on the Bayesian information criterion (BIC), which identified GTR+F + R4 as the best-fitting model. Branch support was assessed using 1,000 bootstrap replicates. The final molecular phylogenetic tree was visualized using iTOL (Letunic and Bork 2021).

## 3. Results

**Figure 2.**
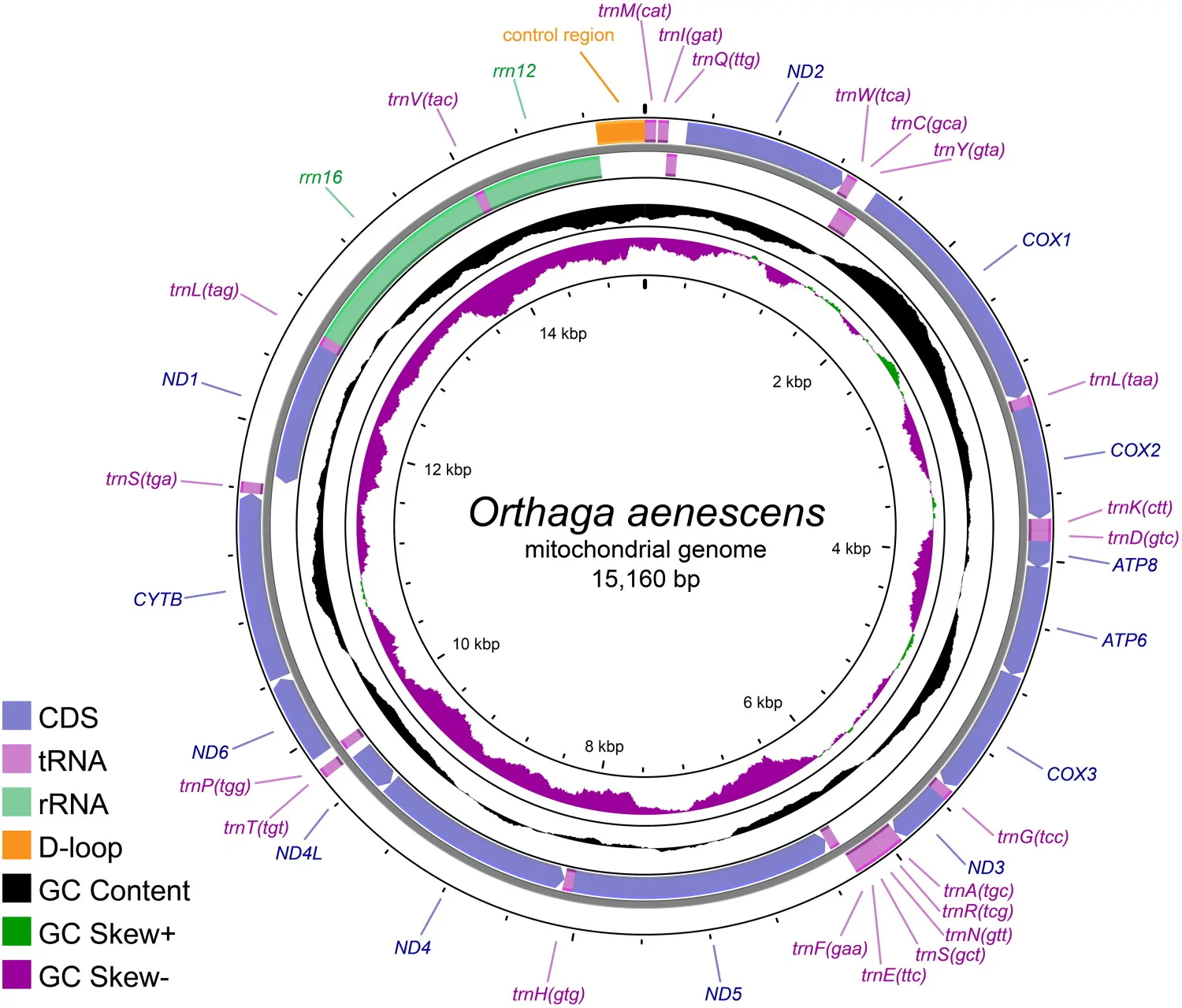
Circular map of the complete mitochondrial genome of *O. aenescens.* Genes drawn outside the outer circle were transcribed clockwise, and those drawn inside were transcribed counter-clockwise. The differently colored legend in the bottom left corner represented genes with different functions. The black inner circle displayed the GC content of the mitochondrial genome, and the innermost ring showed the GC-skew across the mitochondrial genome. The Central scale bar indicates the absolute physical length in base pairs. GC content was calculated using a sliding window of 1,000 bp with a step size of 1 bp, whereas GC skew was calculated using a sliding window of 500 bp with a step size of 1 bp.

The complete mitogenome of *O. aenescens* was a circular molecule of 15,160 bp (GenBank accession number: OR881393), with an average coverage depth of 1956.92× (Figure S1). The *O. aenescens* mitogenome contained 37 typical mitochondrial genes, which were identical in gene arrangement to those of other pyraloid species, including 13 protein-coding genes (PCGs), 22 transfer RNA genes (tRNAs), and 2 ribosomal RNA genes (rRNAs), as well as a control region (Figure 2). Of the 37 genes, 23 genes (9 PCGs and 14 tRNAs) were located on the majority strand, while the remaining 14 genes (4 PCGs, 8 tRNAs, and 2 rRNAs) were located on the minority strand. The base composition was strongly biased toward adenine and thymine, with an A + T content of 80.7%. The AT‑skew was slightly negative (−0.031), and the GC‑skew was also negative (−0.183).

The total length of the 13 PCGs was 11,181 bp, representing 73.8% of the entire mitogenome. Except for *COX1*, which used CGA as the start codon, all other PCGs began with typical ATN initiation codons. Canonical stop codons TAA and TAG were found in nine PCGs: TAA in *ATP8*, *ATP6*, *COX3*, *CYTB*, *ND1*, *ND2*, *ND4L*, and *ND6*, and TAG in *ND3*, while the remaining PCGs ended with an incomplete stop codon (a single T). In total, 28 bp of gene overlap was distributed across 9 positions, with the maximum overlap of 7 bp occurring between *ATP8* and *ATP6*. The intergenic spacers together measured 106 bp, varying in length from 1 to 46 bp. Among them, the longest spacer was situated between the *trnQ(ttg)* and *ND2* genes.

**Figure 3.**
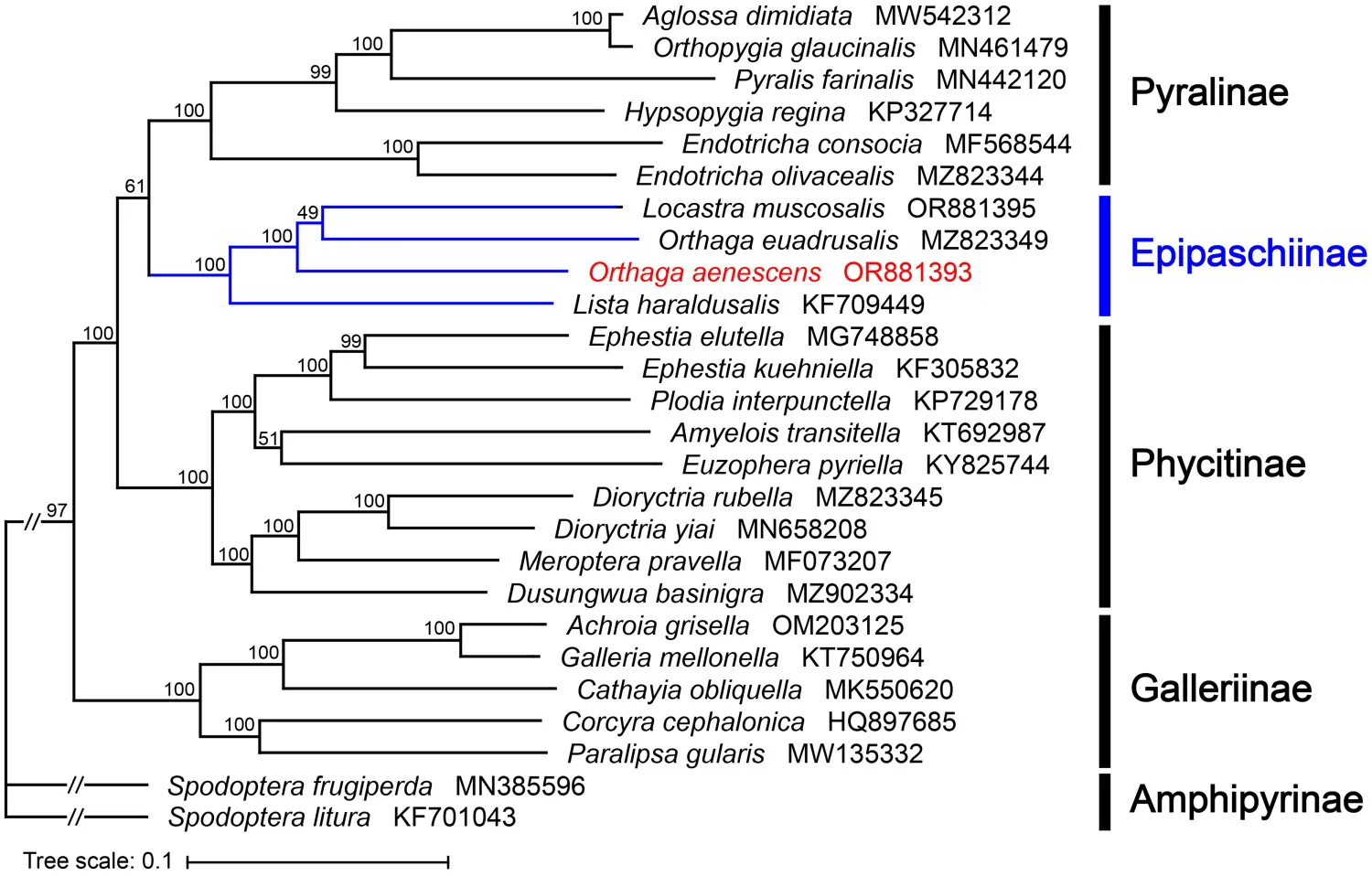
Maximum-likelihood phylogenetic tree inferred from the concatenated nucleotide alignments of 13 mitochondrial PCGs. The values at the nodes indicate bootstrap support values. The scale bar indicates substitutions per site. Long basal and outgroup branches are truncated, as shown by the diagonal break marks. Alphanumeric terms indicate GenBank accession numbers. *Orthaga aenescens* OR881393 is highlighted in red. The subfamily Epipaschiinae is labeled in blue, and all branches within this clade are shown in the same color. The following sequences were used to infer the tree: *Aglossa dimidiata* MW542312 (Hu et al. 2021), *Orthopygia glaucinalis* MN461479 (Mao et al. 2019), *Pyralis farinalis* MN442120 (Mao et al. 2019), *Hypsopygia regina* KP327714 (Zhu et al. 2018), *Endotricha consocia* MF568544 (Zhu et al. 2018), *Endotricha olivacealis* MZ823344 (Liu et al. 2021b), *Locastra muscosalis* OR881395 (Wang et al. 2024), *Orthaga euadrusalis* MZ823349 (Liu et al. 2021b), *Lista haraldusalis* KF709449 (Ye et al. 2015), *Ephestia elutella* MG748858 (Liu et al. 2018), *Ephestia kuehniella* KF305832 (Traut et al. 2013), *Plodia interpunctella* KP729178 (Liu et al. 2016), *Amyelois transitella* KT692987 (Chang and Shen 2016), *Euzophera pyriella* KY825744 (Yang et al. 2017), *Dioryctria rubella* MZ823345 (Liu et al. 2021b), *Dioryctria yiai* MN658208 (Wu et al. 2020), *Meroptera pravella* MF073207 (Living Prairie Mitogenomics Consortium 2017), *Dusungwua basinigra* MZ902334 (Liu et al. 2021b), *Achroia grisella* OM203125 (Liu et al. 2022), *Galleria mellonella* KT750964 (Park et al. 2017), *Cathayia obliquella* MK550620 (Roh et al. 2020), *Corcyra cephalonica* HQ897685 (Wu et al. 2012), *Paralipsa gularis* MW135332 (Guo et al. 2021), *Spodoptera frugiperda* MN385596 (Seo et al. 2019) (outgroup), *Spodoptera litura* KF701043 (Li et al. 2015) (outgroup).

The phylogenetic relationships among the subfamilies within Pyralidae were resolved as follows: (Galleriinae+(Phycitinae +(Epipaschiinae + Pyralinae))) (Figure 3). The newly sequenced species *O. aenescens* belonged to the subfamily Epipaschiinae, which formed a well-supported clade with high bootstrap support values (BSV = 100). Furthermore, *O. aenescens* was found to be closely related to *O. euadrusalis* and *Locastra muscosalis*.

## 4. Discussion and conclusion

The mitogenome of *O. aenescens* contained 37 mitochondrial genes typical of insects, and its gene organization was identical to that of other reported *Orthaga* mitogenomes. The base composition revealed that T was the most prevalent base (39.1%), while G was the least common (7.9%), resulting in a highly biased A + T content of 80.7%. This A + T content was slightly higher than that of other reported *Orthaga* species, such as *O. olivacea* at 79.0% and *O. euadrusalis* at 80.2% (Yang et al. 2020; Liu et al. 2021b), and equal to that of *O. achatina* (Liu et al. 2021a).

All PCGs in the mitogenome started with typical ATN codons, with the exception of *COX1*, which used CGA as the start codon. The canonical termination codons (TAA or TAG) were observed in nine PCGs (TAA for *ATP8*, *ATP6*, *COX3*, *CYTB*, *ND1*, *ND2*, *ND4L*, and *ND6*, TAG for *ND3*), while the remaining PCGs terminated with a single T. The longest overlapping region, consisting of 7 bp, was located between *ATP8* and *ATP6*, containing the motif ‘ATGATAA,’ which had been reported in several sequenced lepidopteran species (Pan et al. 2008; Zhu et al. 2013).

Phylogenetic analysis confirmed that *O. aenescens* belonged to the subfamily Epipaschiinae, showing the closest genetic relationship with *O. euadrusalis* and *L. muscosalis* in the present dataset. The newly characterized mitogenome provides a useful genetic resource for future molecular and taxonomic studies of Epipaschiinae.

## Supplementary Material

Supplemental Material.docx

## Data Availability

The genome sequence data that support the findings of this study are openly available in GenBank of NCBI at https://www.ncbi.nlm.nih.gov/ under the accession number OR881393. The associated BioProject, BioSample, and Sequence Read Archive (SRA) numbers are PRJNA1045995, SAMN38468359, and SRR27050911, respectively.
