# Supplementary material for: The complete mitochondrial genome and phylogenetic position of Orthaga aenescens Moore, 1888 (Lepidoptera: Pyralidae)
Source: Mitochondrial DNA B Resour. 2026 Aug 24;11(9):1064–8. doi: 10.1080/23802359.2026.2722723 (PMC13508489; doi:10.1080/23802359.2026.2722723)
Supplement: Supplementary file 1 [file TMDN_A_2722723_SM2698.docx]

Supplemental material for

**The complete mitochondrial genome and phylogenetic position of *Orthaga aenescens* Moore, 1888 (Lepidoptera: Pyralidae)**

Laiyou Wang^a^ (https://orcid.org/0000-0001-7793-3325), Jiali Liu^b^ (https://orcid.org/0009-0005-9560-1179), Shuyu Hui^b^ (https://orcid.org/0009-0009-3254-2940), Hua Rong^b*^ (https://orcid.org/0009-0002-6653-8404), and Yu Wang^a,b*^ (https://orcid.org/0009-0008-9251-344X)

^a^ School of biological and chemical engineering, Nanyang Institute of Technology, Nanyang, China

^b^ College of bioscience and engineering, Jiangxi Agricultural University, Nanchang, China

CONTACT:

Hua Rong, ronghua@jxau.edu.cn. College of bioscience and engineering, Jiangxi Agricultural University, Nanchang 330045, China

Yu Wang, wangyu@jxau.edu.cn. School of biological and chemical engineering, Nanyang Institute of Technology, Nanyang 473004, China

Figure S1. Sequencing depth and coverage of the mitochondrial genome of *Orthaga aenescens*. The X-axis represents the nucleotide positions along the mitochondrial genome, and the Y-axis shows the coverage depth at each position. The mitochondrial genome is 15,160 bp in length, with the coverage depth ranging from 700× to 2804×, yielding an average coverage depth of 1956.92×.
